# 4-HNE Increases Intracellular ADMA Levels in Cultured HUVECs: Evidence for miR-21-Dependent Mechanisms

**DOI:** 10.1371/journal.pone.0064148

**Published:** 2013-05-22

**Authors:** Lei Chen, Ji-Peng Zhou, Da-Bin Kuang, Jie Tang, Yuan-Jian Li, Xiao-Ping Chen

**Affiliations:** 1 Department of Pharmacology, School of Pharmaceutical Science, Central South University, Changsha, Hunan, China; 2 Pharmacogenetics Research Institute, Institute of Clinical Pharmacology, Central South University, Changsha, Hunan, China; National Institutes of Health, United States of America

## Abstract

**Objective:**

To investigate whether 4-hydroxynonenal (4-HNE) regulates asymmetric dimethylarginine (ADMA) metabolism through pathway independent of direct adduct formation with ADMA metabolizing enzyme and the involvement of microRNA (miRNA) miR-21 in human umbilical venous endothelial cells (HUVECs).

**Methods:**

Cultured HUVECs were treated with 4-HNE (at concentrations of 1, 5, and 10 µM, respectively) or 1‰ DMSO (vehicle control) for 24 h. MiR-21 inhibitor (final concentration of 100 nM) was transfected at 1 h before 4-HNE treatment. HUVECs were also transfected with miR-21 (at concentrations of 50 nM and 100 nM) and cultured for 12, 24, and 48 h, respectively. *DDAH* mRNA and miR-21 expression in the HUVECs were determined by semi-quantitative real time PCR. DDAH1 and DDAH2 protein expression were analyzed by Western blot. ADMA in the cell medium and cell lysates were analyzed by ELISA. ADMA metabolizing activity of the cell lysates was also determined.

**Results:**

MiR-21 decreased *DDAH1* and *DDAH2* expression and ADMA metabolic activity significantly, while increased intracellular ADMA accumulation significantly in HUVECs. 10 µM 4-HNE treatment for 24 h increased the expression of miR-21 and intracellular ADMA concentration, decreased the expression of DDAH1/2 mRNA and protein, decreased ADMA metabolizing activity of the cell lysates significantly. MiR-21 inhibitor reversed the inhibitory effects of 4-HNE on DDAH1 expression completely, and partially reversed the changes in ADMA metabolizing activity and intracellular ADMA accumulation challenged by 10 µM 4-HNE.

**Conclusion:**

4-HNE down-regulates DDAH1 expression and increases intracellular ADMA accumulation in HUVECs through a miR-21-dependent mechanism.

## Introduction

The endogenous NO synthase (NOS) inhibitor asymmetric dimethylargiline (ADMA) can decrease NO production through inhibiting NOS competitively and is proved to play important roles in the development of diseases such as hypertension, atherosclerosis (AS), coronary arterial disease, stroke, insulin resistance, and diabetes mellitus [1], [2], [3]. ADMA has been regarded as a novel and independent predictor of cardiovascular events in recent years [4]. Dimethylarginine dimethylaminohydrases (DDAHs) are key enzymes involved in the inactivation of ADMA in the body. The DDAH/ADMA/NOS pathway has become a potential target of drug discovery for cardiovascular diseases. Two isoforms of the DDAHs, i.e DDAH1 and DDAH2, are found in human. Though there is evidence that DDAH2 is involved in ADMA metabolism [5], DDAH1 is proved to be the critical enzyme responsible for ADMA metabolism *in vivo* [6].

**Figure 1:**
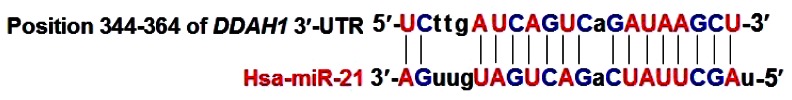
The prediction of miR-21 binding site in *DDAH1* 3′-UTR with the online software Target Scan.

MicroRNAs (miRNAs) are a class of highly conserved short non-coding RNAs which regulate a majority of the human genome at post-transcriptional level [7]. It is recognized that miRNAs regulate not only normal cellular functions such as cell proliferation and differentiation, but also contribute to the development of human diseases such as cancer and cardiovascular diseases [8], [9]. MiR-21 is one of the miRNAs that contributes to the development of cardiovascular diseases such as cardiac hypertrophy and cardiovascular remodeling. An increase in circulating miR-21 levels is associated with kidney fibrosis [10]. MiR-21 also plays important roles in the ischemic heart with resveratrol [11]. A 4.6-fold increase in miR-21 expression in AS plaque is observed [12]. By using microarray analyses, Fleissner and colleagues find that miR-21 is one of the 16 miRNAs up-regulated by ADMA treatment in circulating angiogenic progenitor cells (APCs) [13]. Evidence shows that ADMA impaires the migratory capacity of APCs through increasing miR-21 expression, repressing the expression of its target sprouty-2, as well as increasing reactive oxygen species (ROS) formation [13]. In addition, in patients with coronary artery disease demonstrating high ADMA plasma levels, an elevation in miR-21 expression in APCs is observed [13]. Interestingly, we observed that *DDAH1* is predicted to be a potential miR-21 target (Figure 1). Therefore, we raised the hypothesis that there might be a positive feedback loop between ADMA and miR-21 in accelerating the progress of cardiovascular diseases.

4-Hydroxynonenal (4-HNE) is a major active product formed following lipid peroxidation. 4-HNE is highly lipophilic and can interfere with the functions of proteins by adduct-forming capacity with macromolecules [14]. Physiological levels of 4-HNE in human plasma ranged in 0.3∼1.0 µM. However, under pathophysiological conditions, the levels may increase to 10 µM or even higher. Double-edged sword effects for 4-HNE are observed: induction of the expression of antioxidant enzymes at physiological levels [15]; inhibition or inactivation of enzymes, membrane proteins and cytoskeletal proteins by forming 4-HNE-protein adducts at pathophysiological levels [14]. The molecule can also affect signal transduction pathways such as the insulin-dependent Akt signaling [16], [17] and the PI3K/Akt pathway [18]. Increased levels of 4-HNE in plasma and biological fluids are observed in many human diseases including atherosclerosis [19], and 4-HNE can also contribute to the development of atherosclerosis and related diseases. Evidence shows that 4-HNE can inhibit DDAH1 activity and decrease NO generation in cultured bovine aortic endothelial cells in a dose-dependent manner through formation of Michael adducts on His 173 in DDAH1 [20], [21]. However, it is unknown whether 4-HNE regulates DDAH activity and ADMA levels through other mechanisms.

In this study, we determined whether 4-HNE could influence intracellular ADMA levels through regulating DDAH1 expression and the contribution of miR-21 in cultured HUVECs. We found that both 4-HNE and miR-21 decreased DDAH1 expression and ADMA metabolizing activity in HUVECs. Meanwhile, 4-HNE up-regulated miR-21 expression, and the inhibitory effects of 4-HNE on DDAH1 expression was reversed by miR-21 inhibitor.

## Methods

### 4-HNE Treatment and miR-21 Mimic Transfection of HUVECs

The HUVECs cell line was purchased from ATCC CRL-1730. 4-HNE (10 mg/ml in 100% ethanol) was purchased from Cayman Chemical Co. (Ann Arbor, MI, USA), dried under a N_2_ flow and redissolved in 1% DMSO. Cells (1×10^5^/mL) were seeded into 6-well plates and cultured in DMEM medium containing 10% fetal calf serum (FCS) in a humidified atmosphere under 5% CO_2_ for 24 h. Confluent cells were synchronized with 1% FCS for 24 hours, and were then treated with various concentration of 4-HNE (1 µM, 5 µM, and 10 µM) or vehicle (1‰ DMSO) for 24 h. To investigate the effect of miR-21 on DDAH expression, HUVECs were seeded into 6-well plates and cultured to 95% confluence. Cells were then washed with PBS and cultured in DMEM medium containing 10% FCS. Has-miR-21 mimic (50 nM and 100 nM) and/or inhibitor (100 nM) (RiboBio, China) were transfected by using the lipofectamine 2000 reagents (Fermentas, R0531). Cells and the medium were harvested at 12 h, 24 h, and 48 h, respectively, after miR-21 transfection. To investigate whether miR-21 inhibitor can reverse the 4-HNE induced increase in DDAH1 expression, has-miR-21 inhibitor was also transfected at 1 h before the addition of 4-HNE (10 µM). The medium was collected to detect ADMA concentration. Cells were harvested and the RNA and protein samples were extracted.

### Determination of mRNA, pri-miRNA and miRNA Expression by Semi-quantitative Real-time PCR (RT-PCR)

Total RNA was extract from HUVECs using Trizol reagent according to the manufacturer’s protocol (Takara, Japan). RNA samples of 0.5 ug from each sample were used for reverse transcription. Semi-quantitative analysis of pri-miR-21, mature miR-21, *DDAH*1 and *DDAH2* mRNA expression was detected by SYBR®Green chemistry on an ABI 7300 real-time PCR system. Endogenous small nuclear RNA U6, 5S and *GAPDH* mRNA were determined to normalize miR-21, pri-miR-21, and *DDAH1/2* mRNA expression, respectively. The primers used were as follows: *DDAH1*: 5′-GCCTGATGACATAGCAGCAA-3′ (sense) and 5′-CCATCCACCTTTTCCAGTTC-3′ (antisense); *DDAH2*: 5′-ACAAGGACCCCCGCTAAAA-3′ (sense) and 5′-AAGGGAGTCCCCGTCTTCAA-3′ (antisense); *GAPDH*: 5′-CTGCACCACCAACTGCTTAG-3′ (sense) and 5′-AGGTCC-ACCACTGACACGTT-3′ (antisense). Pri-miR-21: 5′-GCCACCACACCAGCTAATTT-3′ (sense) and 5′-CTGAAGTCGCCATGCAGATA-3′ (antisense); 5S: 5′-GCCCGATCTCGTCTGATCT-3′ (sense) and 5′-AGCCTACAGCACCCGGTATT-3′ (antisense). MiR-21 and the small nuclear RNA U6 quantitative PCR primers were purchased (RiboBio, China). Expression of miR-21 was normalized for endogenous U6. Expression of pri-miR-21 was normalized for endogenous 5S. PCR conditions were as follows: an initial denaturation at 95°C for 30s, followed by 40 cycles of denaturation at 95°C for 5s, and annealing at 60°C for 30s. All amplification reactions were performed in triplicate, and the averages of the threshold cycles were used to interpolate curves using the 7300 System SDS Software. Results were expressed as the ratio of *DDAH* mRNA to *GAPDH* mRNA, miR-21 to U6 RNA, and pri-miR-21 to 5S RNA, respectively.

### Western Blot Analysis

HUVECs cells were collected and washed with PBS. Cells were treated with 200 µL of cell lysis buffer at 4°C for 30 min and then centrifuged at 1,2000 rpm for 10 min. The cell lysates were then stored at −80°C until analysis. An aliquot of 60 µg proteins from each sample were subjected to 10% SDS-PAGE and then transferred to PVDF membrane (Millipore Corp). Semi-dried transfer was carried out under 1 mA/cm2 flow in 50 min. The membrane was blocked with 5% non-fat dry milk for 2 h at room temperature, and then incubated with a rabbit monoclonal antibody (1∶1000) against human DDAH1 (Abcam, UK), or a rabbit monoclonal antibody (1∶1000) against human DDAH2 (sigma, USA), or a mouse monoclonal antibody (1∶1000) against human GAPDH (sigma, USA) at room temperature for 1 h, followed by incubation at 4°C for 18 h. The membrane was washed thoroughly and then incubated with a peroxidase-conjugated mouse anti-rabbit or goat anti-mouse secondary antibody (1∶5000) (Santa Cruz, USA) for 1 h at room temperature. The membrane was washed, and DAB Horseradish Peroxidase Color Development Kit was used for develop (beyotime, China). The band intensities were semi-quantified, DDAH1 or DDAH2 protein levels were expressed as the ratio of the band intensities of DDAH1 or DDAH2 to the endogenous control GAPDH.

### Determination of ADMA in Cell Medium and Cell Lysates by ELISA

Cells and cellular medium were collected after treatment. Cells were washed with PBS, and then treated with 200 µL of cell lysates buffer at 4°C for 30 min, centrifuged at 1,2000 rpm for 10 min. Protein concentration of the cell lysates were determined. Cell Lysates and cellular medium were then stored at −80°C. An aliquot of 10 µL cell lysates was used to detect intracellular ADMA concentration. Human ADMA ELISA kit was used to analysis ADMA concentration in cell medium and the lysates according to the manufacturer’s protocol (CUSABIO, China). ADMA contents in the cell lysis were normalized by protein content.

### Determination of ADMA Metabolizing Activity in Cell Lysates

DDAH activity was determined by measurement of the amount of ADMA metabolized by the cell lysates as described elsewhere [22]. An aliquot of 80 µL cell Lysates from each well was added into two separate Eppendorf tubes (on ice), followed by the addition of 40 µL ADMA (1 mmol/L). Then, 40 µL 30% 5-sulfosalicylic acid was immediately added into one tube to inactivate DDAH enzymes; the other tube was incubated at 37°C for 2 hours before the addition of 5-sulfosalicylic acid. The tubes were then centrifuged and ADMA concentrations in the supernatant were measured by ELISA assay kit (CUSABIO, China). Difference in ADMA content between the 2 tubes indicated ADMA metabolized. ADMA metabolizing activity was expressed as amount of ADMA metabolized in 1 min by 1 g protein (µmol/min/g protein).

### Data Analysis

Data were expressed as mean ± SEM. All statistical analysis were performed using the software SPSS, version 11.5 (SPSS Inc., Chicago, IL, USA). Differences among groups were compared by using univariate analysis of variance (ANOVA), and comparisons between two groups were analyzed by the LSD test. Two-tailed *p*<0.05 was considered to be statistically significant.

## Results

### MiR-21 Inhibited DDAH Expression and Increased Intracellular ADMA Concentration in Cultured HUVECs

**Figure 2:**
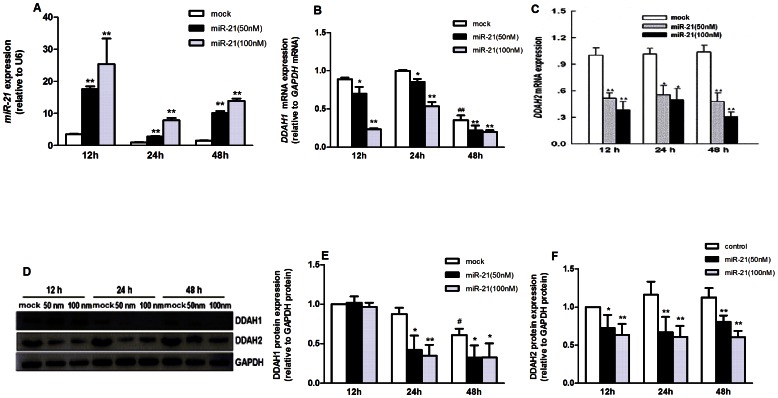
Effects of miR-21 transfection on DDAH expression and intracellular ADMA level in cultured HUVECs. Data were expressed as means±SEM (n = 3). **A.** Expression of miR-21 in HUVECs at different time point after miR-21 transfection. **p<0.01, compared with the control group at each time point; **B.** Relative expression of *DDAH1 mRNA* in HUVECs after miR-21 transfection. ^*^ *p*<0.05, ^**^ *p*<0.01, as compared with the control at corresponding time point; ^##^ *p*<0.01, as compared with the control group at 12 h; **C.** Expression of *DDAH2 mRNA* in HUVECs after miR-21 transfection. ^*^ *p*<0.05, ^**^ *p*<0.01, as compared with the control group at corresponding time point. **D.** Western blot analysis of DDAH1 and DDAH2 expression after miR-21 transfection. **E** and **F.** Semi-quantitative expression of DDAH1 and DDAH2 protein expression in HUVECs after miR-21 transfection. ^*^ *p*<0.05. ^**^ *p*<0.01, as compared with control group at corresponding time point; ^#^ *p*<0.05, as compared with the control group at 12 h.

Transfection of miR-21 at concentrations of both 50 nmol/L and 100 nmol/L increased miR-21 expression in HUVECs significantly since 12 h after transfection (*p*<0.01, respectively). The increases in intracellular miR-21 expression decreased gradually but were still significant at 24 h and 48 h, respectively, after transfection (*p*<0.01, respectively, Figure 2A). Both 50 and 100 nmol/L miR-21 decreased *DDAH1* and *DDAH2* mRNA expression significantly at all three time points after transfection (*p*<0.05, respectively, Figure 2B and 2C). When DDAH protein expression was analyzed, both concentrations of miR-21 decreased DDAH1 expression at 24 h and 48 h (*p*<0.05, respectively, Figure 2D and 2E) but not at 12 h after transfection (Figure 2D and 2E). DDAH2 protein expression was decreased at all three time points after miR-21 transfection (*p*<0.05, respectively, Figure 2D and 2F). As compared with the control group at 12 h and 24 h, both *DDAH1* mRNA and protein expression were decreased significantly in the control group at 48 h (*p*<0.05, respectively).

**Figure 3:**
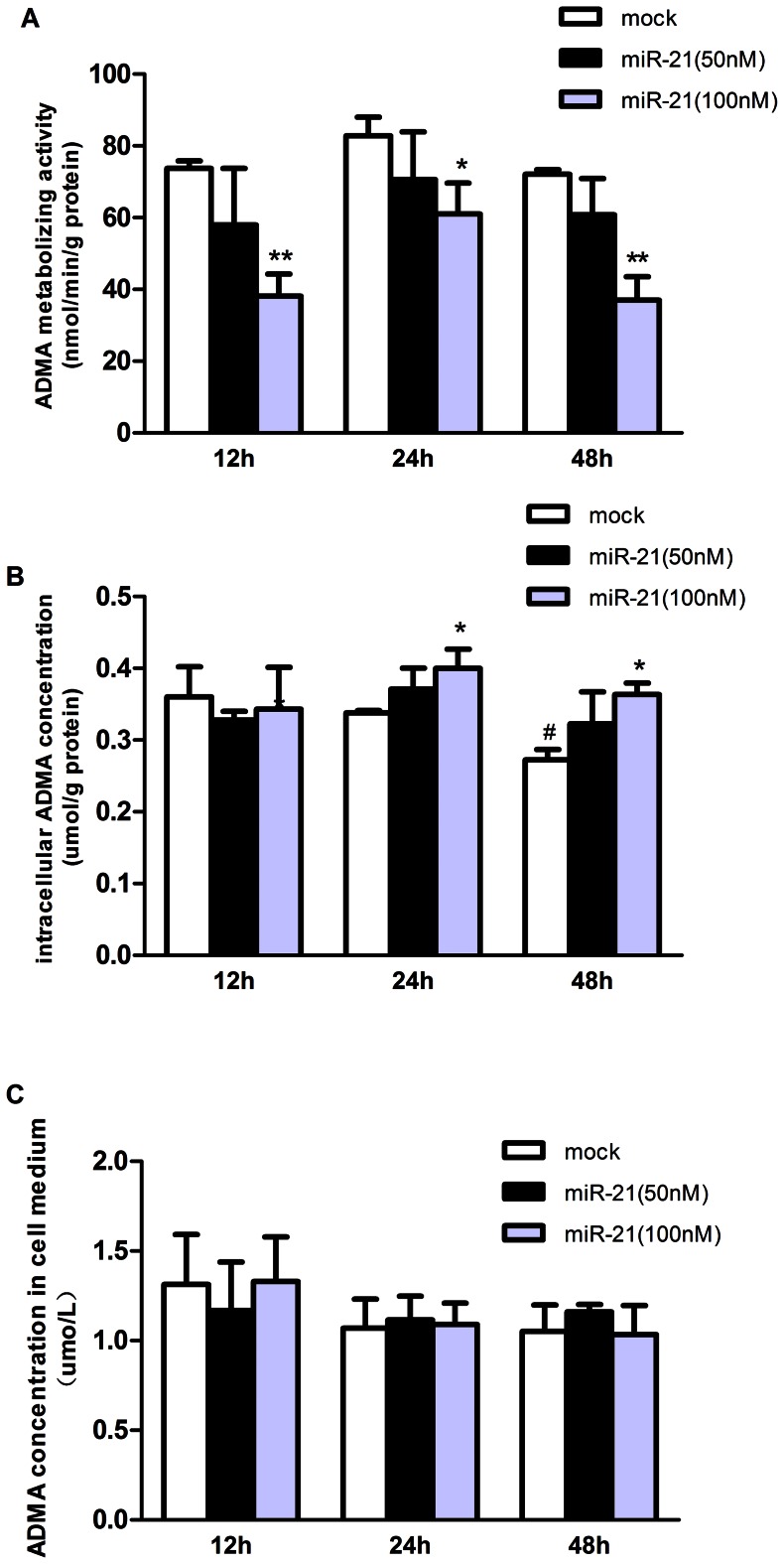
Influence of miR-21 transfection on intracellular ADMA metabolic activity and ADMA concentrations in HUVECs. Data were expressed as means±SEM (n = 3). **A.** ADMA metabolic activity of the cell lysates after miR-21 transfection. *p<0.05, **p<0.01, as compare with control group at corresponding time point; **B.** Intracellular ADMA concentration after miR-21 transfection. *p<0.05, as compare with control group at corresponding time point; ^#^ *p*<0.05 compare with control group at 12 h; **C.** Concentration of ADMA in cellular medium after miR-21 transfection.

ADMA metabolizing activity of the cell lysates was decreased significantly at all three time points after 100 nmol/L miR-21 transfection (*p*<0.05 for 24 h, and *p*<0.01 for 12 and 48 h, respectively, Figure 3A). Intracellular ADMA concentration was increased significantly at 24 and 48 h (*p*<0.05, respectively) but not at 12 h (*p*>0.05) after 100 nmol/L miR-21 treatment (Figure 3B). 50 nmol/L miR-21 treatment did not affect either the intracellular ADMA concentration or ADMA metabolizing activity of the cell lysates (Figure 3A and 3B). MiR-21 transfection showed no effects on ADMA concentration in cell medium. As compared with the control group at 12 h and 24 h, intracellular ADMA concentration was decreased significantly in the control group at 48 h (*p*<0.05, Figure 3B).

### 4-HNE Decreased DDAH1/2 Expression and Increased Intracellular ADMA Concentration in Cultured HUVECs

**Figure 4:**
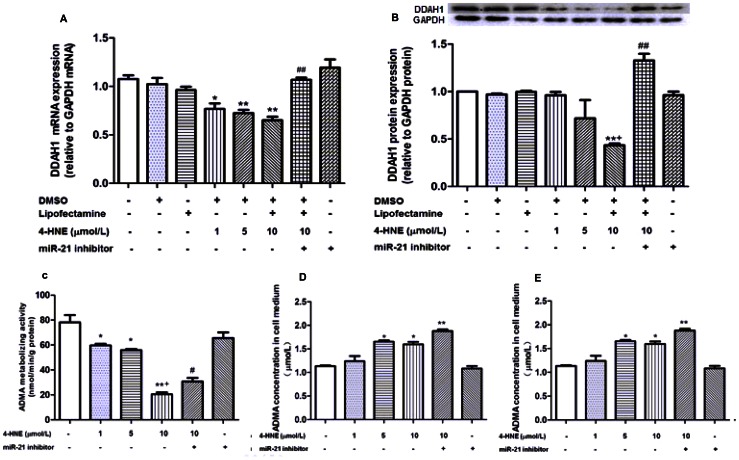
Influence of 4-HNE treatment on ADMA metabolic activity and DDAH1 expression after miR-21 inhibitor transfection. Data were expressed as means±SEM (n = 3). **A.** Relative *DDAH1 mRNA* expression with or without (control) 4-HNE treatment for 24 hours, ^*^ *p*<0.05,^ **^ *p*<0.01, as compared with the control group; ^##^ *p*<0.01, as compared with 10 µm 4-HNE group; **B.** DDAH1 protein expression in HUVECs after 4-HNE treatment. **p<0.01, as compared with the control group; ^+^ *p*<0.05, as compared with 1 µM and 5 µM 4-HNE treatment group; ^##^ *p*<0.01, as compared with 10 µM 4-HNE group; **C.** Effect of 4-HNE treatment on ADMA metabolic activity of the cell lysates. *p<0.05, **p<0.01, as compared with the control group; ^+^ *p*<0.05, as compared with 1 µM and 5 µm 4-HNE group; ^#^ *p*<0.05, as compared with 10 µm 4-HNE group. **D.** Intracellular ADMA levels after 4-HNE treatment.^ *^ *p*<0.05, **p<0.01, as compared with the control group; ^#^ *p*<0.05, as compared with 4-HNE (10 µm) group; **E.** Concentration of ADMA in cellular medium after 4-HNE addition, *p<0.05, as compared with the control group.

**Figure 5:**
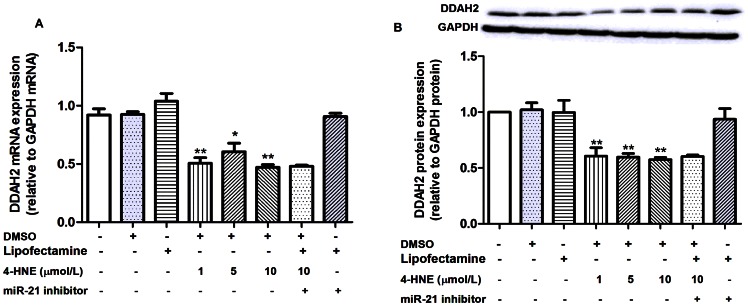
Influence of 4-HNE treatment on DDAH2 expression and the effect of miR-21 inhibitor in HUVECs. Data were expressed as means±SEM (n = 3). **A.**Influence of 4-HNE treatment alone or combined with miR-21 inhibitor transfection on DDAH2 mRNA expression in HUVECs, ^*^ *p*<0.05.^ **^ *p*<0.01, as compared with the control group.; **B.** Influence of 4-HNE treatment alone or combined with miR-21 inhibitor transfection on DDAH2 protein expression in HUVECs.

Treatment of HUVECs with various concentrations of 4-HNE for 24 hours decreased *DDAH1* mRNA expression significantly (*p*<0.05 for 1 µmol/L; *p*<0.01 for 5 µmol/L and 10 µmol/L, respectively, Figure 4A). The degree of decrease in *DDAH1* mRNA expression was more obvious in 5 µmol/L and 10 µmol/L 4-HNE treated as compared with 1 µmol/L 4-HNE treated cells (*p*<0.05, respectively, Figure 4A). Significant decrease in DDAH1 protein expression was observed in cells treated with 10 µmol/L 4-HNE (*p*<0.01, Figure 4B) but not with 1 µmol/L and 5 µmol/L 4-HNE. Both *DDAH2* mRNA and protein expression were decreased by 4-HNE treatment at all three concentrations (*p*<0.01, Figure 5). DMSO (solvent control) or lipofectamine 2000 (transfection reagent control) alone had no effect on either *DDAH1* mRNA or protein expression (Figure 4).

Intracellular ADMA metabolizing activity was decreased while ADMA concentration was increased significantly by 4-HNE treatment at all three concentrations (*p*<0.05, Figure 4C and 4D). The degree of decrease in ADMA metabolizing activity was more obvious for the 10 µmol/L as compared with both 1 µmol/L and 5 µmol/L 4-HNE treated cells (*p*<0.01, Figure 4C). The increase in intracellular ADMA concentration was comparable among the different concentration of 4-HNE treated groups (Figure 4D). ADMA concentration in cell culture was increased by 5 µmol/L and 10 µmol/L 4-HNE treatment (*p*<0.05, respectively, Figure 4C).

### 4-HNE at High Concentration Up-regulated pri-miR-21 and miR-21 Expression in HUVECs

**Figure 6:**
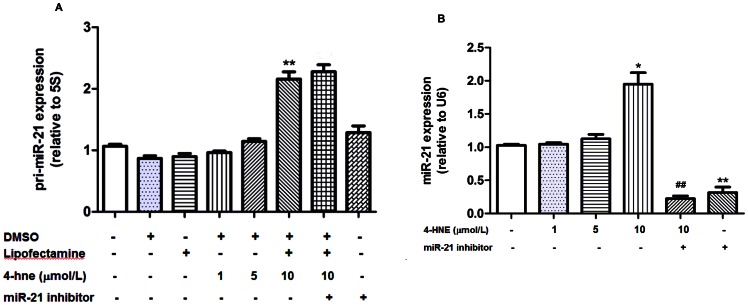
Effects of 4-HNE treatment on pri-miR-21 and miR-21 expression in cultured HUVECs. Data were expressed as means±SEM (n = 3). **A.** Effect of 4-HNE treatment on pri-miR-21 expression. ^*^ *p*<0.05.^ **^ *p*<0.01, as compared with the control group; ^##^ *p*<0.01, as compared with 4-HNE (10 µm) group; **B.** Effect of 4-HNE treatment on miR-21 expression. ^*^ *p*<0.05.^ **^ *p*<0.01, as compared with the control group.

As shown in Figure 6, both intracellular pri-miR-21 and miR-21 expression were increased significantly by about 2-fold in HUVECs treated with 10 µmol/L 4-HNE (*p*<0.05, Figure 6). Neither 1 µmol/L nor 5 µmol/L 4-HNE treatment affected intracellular miR-21 expression significantly (*p*>0.05, Figure 6). MiR-21 inhibitor (100 nmol/L) significantly decreased the intracellular expression of miR-21 in cells treated with or without 10 µmol/L 4-HNE (*p*<0.01, respectively, Figure 6B). MiR-21 inhibitor (100 nmol/L) did not affect pri-miR-21expression with or without 4-HNE (10 µmol/L) treatment (*p*>0.05, Figure 6A).

### MiR-21 Inhibitor Reversed 4-HNE-induced Decrease in DDAH Expression in HUVECs

To make clear whether the 4-HNE-induced decrease in DDAH1 expression was mediated by miR-21, HUVECs were treated with miR-21 inhibitor (100 nmol/L) 30 min before the addition of 10 µmol/L 4-HNE. As compared with 10 µmol/L 4-HNE treated group, cells in the 4-HNE (10 µmol/L)+miR-21 inhibitor group showed significantly increased DDAH1 mRNA and protein expression (*p*<0.05, respectively, Figure 4A and 4B), significantly increased intracellular ADMA metabolizing activity (*p*<0.05, Figure 4C), and significantly decreased intracellular ADMA concentration (*p*<0.05, Figure 4C). DDAH1 mRNA and protein levels in 4-HNE (10 µmol/L)+miR-21 inhibitor group were comparable to those in the control group. ADMA concentration in cell medium was comparable in 4-HNE (10 µmol/L) and 4-HNE (10 µmol/L)+miR-21 inhibitor treated cells (*p*>0.05, Figure 4E). The 4-HNE+miR-21 inhibitor group showed comparable DDAH2 mRNA and protein level with the 4-HNE (10 µmol/L) group (Figure 5). MiR-21 inhibitor alone showed no effect on either DDAH1 expression or intracellular ADMA metabolizing activity significantly (Figure 4A, 4B, 4C).

## Discussion

In this study, we observed the influences of 4-HNE and miR-21 on DDAH/ADMA system for the first time. We found that high concentration 4-HNE increased intracellular miR-21 expression and ADMA accumulation, decreased DDAH1 expression and ADMA metabolizing activity in cultured HUVECs. We also observed that overexpression of miR-21 by miR-21 mimic transfection down-regulated both DDAH1 and DDAH2 expression, decreased intracellular ADMA metabolizing activity and increased intracellular ADMA concentration in HUVECs. In addition, miR-21 inhibitor reversed the 4-HNE-induced decrease in DDAH1 expression, and partially reversed the 4-HNE induced increase in intracellular ADMA accumulation and decrease in ADMA metabolic activity.

**Figure 7:**
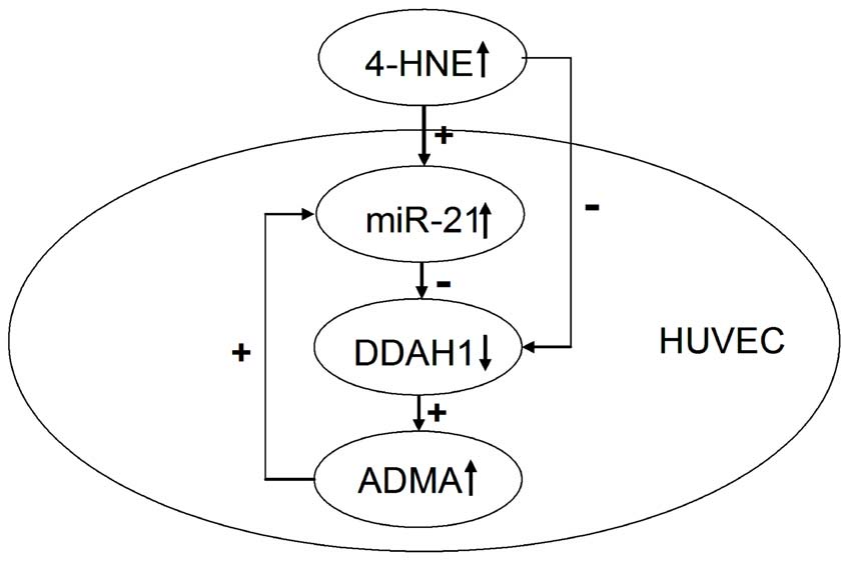
Regulation of 4-HNE on DDAH1/ADMA and the positive feed-back loop between DDAH1/ADMA pathway and miR-21.

ADMA, miR-21 and 4-HNE are all factors contribute to the development of CVDs especially for atherosclerosis. Previous study has shown that ADMA impairs the migratory ability of APCs through up-regulating miR-21 expression [13]. In our study, we found a potential miR-21 binding element (position 344∼364) in *DDAH1* 3′-UTR, and observed that overexpression of miR-21 by lipofectamine mediated transfection at concentrations of both 50 nmol/L and 100 nmol/L down-regulated both DDAH1 and DDAH2 expression in cultured HUVECs. However, only 100 nmol/L miR-21 transfection affected intracellular ADMA metabolizing activity and intracellular ADMA level. The inhibition of *DDAH1* mRNA expression occurred at 12 h while inhibition of DDAH1 protein expression was observed at 24 h after miR-21 transfection, suggesting a time delay in the inhibition of DDAH1 expression by miR-21. It is known that miRNAs act through translational repression or degradation of target mRNA at post-transcriptional level [23]. Though we failed to perform the reporter gene assay that may provide more direct evidence to confirm whether *DDAH1* is the target for miR-21, our observation of the delay between the effects of miR-21 on DDAH1 mRNA and protein expression indicates that miR-21 may act through affecting *DDAH1* mRNA stability. As ADMA can also upregulate miR-21 expression [13], our findings suggest a possible positive feedback regulation loop between miR-21 and the DDAH/ADMA system, which may accelerate the development of AS and related human diseases (Figure 7). MiR-21 is supposed to decrease NO production by increasing intracellular ADMA levels in HUVECs according to our observations. However, a study by Weber et al has shown that miR-21 overexpression increases NO production by promoting eNOS phosphorylation in HUVECs [24]. This controversy should be further studied in future.

Obvious inhibition of DDAH2 mRNA and protein expression by miR-21 was also observed in our study. As no miR-21 recognition site in the *DDAH2* mRNA was observed, the exact mechanism for this inhibition remains unknown. Since the expression of DDAH2 is ROS sensitive [25], while miR-21 can regulate ROS formation by attenuating SOD3 or limiting TNFα production [26], there is the possibility that miR-21 down-regulates DDAH2, and possibly partially for DDAH1, through ROS-dependent pathways. We also observed that miR-21 inhibitor did not reverse 4-HNE induced decrease in DDAH2 expression like DDAH1, which also supports our findings that miR-21 may inhibit DDAH2 through indirect mechanisms.

It is interesting that 50 nM miR-21 transfection reduced both mRNA and protein expression of DDAH1 and DDAH2, however, there was just a trend of increased intracellular ADMA level and decreased DMA metabolizing activity of the cell lysates challenged by 50 nM miR-21. The reason for this discrepancy is not known. The intracellular ADMA level is a result of the balance among ADMA production, metabolism and efflux from the intracellular stores. The proteins such as protein argiline methyltransferase (PRMTs) [27], alanine glyoxylate aminotransferase2 (AGXT2) [28], and the cationic amino acid transporter [29] are also involved in the regulation of intracellular ADMA concentration. Therefore, we assume that miR-21 might also regulate the expression or activity of these proteins, and thus compensates for the changes in intracellular ADMA level and metabolizing activity due to DDAH1 inhibition.

4-HNE is considered as a new atherogenic factor, and plasma 4-HNE level is suggested to be potential biomarker for AS related disease [30], [31]. Few mechanisms by which 4-HNE acts through are reported [15], [21]. Evidence has shown that 4-HNE can increase ADMA accumulation in HUVECs by formation of Michael adducts with DDAH1 [21]. In addition to this, we observed for the first time that 4-HNE increases intracellular ADMA accumulation by inhibiting DDAH1 expression, while miR-21 inhibitor completely reversed the inhibitory effects of 4-HNE (10 µM) on DDAH1 expression. This indicates that 4-HNE can also affect DDAH1/ADMA system in a miR-21 dependent pathway. However, it is still not known as to how 4-HNE up-regulates miR-21 expression. It is reported that 4-HNE increases the generation of reactive oxygen species (ROS) by promoting the activity of NADPH oxidase [32]. Activation of the ROS-NF-κB/ERK pathway by 4-HNE is also observed [33]. Interestingly, the same ROS-NF-κB/ERK pathway also mediates up-regulation of miR-21 expression under oxidative stress [34]. It is possible that the 4-HNE induced up-regulation of miR-21 expression observed in our study is mediated by the ROS-NF-κB/ERK pathway. Of course, further studies are needed to verify this hypothesis.

We observed that miR-21 inhibitor only partially reversed ADMA metabolizing activity of the cell lysates and intracellular accumulation of ADMA by 10 µM 4-HNE treatment. These findings indicate that miR-21-independent mechanisms are also involved in the regulation of ADMA metabolism under 4-HNE exposure. Direct inhibition of DDAH1 activity by 4-HNE may account for this phenomenom [21]. We also observed that miR-21 inhibitor further increased ADMA levels in cell medium after 10 µM 4-HNE challenge. There is still no reasonable explanation for this observation. As it is known, the transmembrane transportation (intake and extrusion) of ADMA is mediated by transporters such as cationic amino acid transporter 1 [35], [36]. There is the possibility that miR-21 may also act through affecting ADMA transportation in HUVECs when challenged by 4-HNE.

### Conclusion

In conclusion, we find in this study that 4-HNE can dysregulate intracellular ADMA in HUVECs through miR-21 dependent pathway and involving inhibition of DDAH1 expression. We also suggest a positive regulation loop between miR-21 and the DDAH1/ADMA system. Our findings suggest that intervention with miR-21 expression may provide a new therapeutic target for the treatment of 4-HNE and ADMA related diseases such as atherosclerosis.

## References

[1] Boger RH, Sullivan LM, Schwedhelm E, Wang TJ, Maas R, et al (2009) Plasma asymmetric dimethylarginine and incidence of cardiovascular disease and death in the community. Circulation 119: 1592–1600. 19289633 10.1161/CIRCULATIONAHA.108.838268 PMC2742491

[2] Niebauer J, Maxwell AJ, Lin PS, Wang D, Tsao PS, et al (2003) NOS inhibition accelerates atherogenesis: reversal by exercise. Am J Physiol Heart Circ Physiol 285: H535–H540. 12598230 10.1152/ajpheart.00360.2001

[3] Wilson AM, Shin DS, Weatherby C, Harada RK, Ng MK, et al (2010) Asymmetric dimethylarginine correlates with measures of disease severity, major adverse cardiovascular events and all-cause mortality in patients with peripheral arterial disease. Vasc Med 15: 267–274. 20484311 10.1177/1358863X10364552 PMC3131178

[4] Miyazaki H, Matsuoka H, Cooke JP, Usui M, Ueda S, et al (1999) Endogenous nitric oxide synthase inhibitor: a novel marker of atherosclerosis. Circulation 99: 1141–1146. 10069780 10.1161/01.cir.99.9.1141

[5] Hasegawa K, Wakino S, Tatematsu S, Yoshioka K, Homma K, et al (2007) Role of asymmetric dimethylarginine in vascular injury in transgenic mice overexpressing dimethylarginie dimethylaminohydrolase 2. Circ Res 101: e2–e10. 17601800 10.1161/CIRCRESAHA.107.156901

[6] Wang D, Gill PS, Chabrashvili T, Onozato ML, Raggio J, et al (2007) Isoform-specific regulation by N(G),N(G)-dimethylarginine dimethylaminohydrolase of rat serum asymmetric dimethylarginine and vascular endothelium-derived relaxing factor/NO. Circ Res. 101: 627–635. 10.1161/CIRCRESAHA.107.158915 17673667

[7] Avraham R, Yarden Y (2012) Regulation of signalling by microRNAs. Biochem Soc Trans 40: 26–30. 22260661 10.1042/BST20110623 PMC3621035

[8] Yamakuchi M (2012) MicroRNAs in Vascular Biology. Int J Vasc Med 2012: 794898. 23056947 10.1155/2012/794898 PMC3463915

[9] Saito Y, Saito H (2012) MicroRNAs in cancers and neurodegenerative disorders. Front Genet 3: 194. 23056009 10.3389/fgene.2012.00194 PMC3458258

[10] Glowacki F, Savary G, Gnemmi V, Buob D, Van der Hauwaert C, et al (2013) Increased Circulating miR-21 Levels Are Associated with Kidney Fibrosis. PLoS One 8: e58014. 23469132 10.1371/journal.pone.0058014 PMC3585177

[11] Mukhopadhyay P, Mukherjee S, Ahsan K, Bagchi A, Pacher P, et al (2010) Restoration of altered microRNA expression in the ischemic heart with resveratrol. PLoS One 5: e15705. 21203465 10.1371/journal.pone.0015705 PMC3009730

[12] Raitoharju E, Lyytikainen LP, Levula M, Oksala N, Mennander A, et al (2011) miR-21, miR-210, miR-34a, and miR-146a/b are up-regulated in human atherosclerotic plaques in the Tampere Vascular Study. Atherosclerosis 219: 211–217. 21820659 10.1016/j.atherosclerosis.2011.07.020

[13] Fleissner F, Jazbutyte V, Fiedler J, Gupta SK, Yin X, et al (2010) Short communication: asymmetric dimethylarginine impairs angiogenic progenitor cell function in patients with coronary artery disease through a microRNA-21-dependent mechanism. Circ Res 107: 138–143. 20489163 10.1161/CIRCRESAHA.110.216770

[14] Poli G, Schaur RJ, Siems WG, Leonarduzzi G (2008) 4-hydroxynonenal: a membrane lipid oxidation product of medicinal interest. Med Res Rev 28: 569–631. 18058921 10.1002/med.20117

[15] Ishikado A, Nishio Y, Morino K, Ugi S, Kondo H, et al (2010) Low concentration of 4-hydroxy hexenal increases heme oxygenase-1 expression through activation of Nrf2 and antioxidative activity in vascular endothelial cells. Biochem Biophys Res Commun 402: 99–104. 20920477 10.1016/j.bbrc.2010.09.124

[16] Shearn CT, Fritz KS, Reigan P, Petersen DR (2011) Modification of Akt2 by 4-hydroxynonenal inhibits insulin-dependent Akt signaling in HepG2 cells. Biochemistry 50: 3984–3996. 21438592 10.1021/bi200029w

[17] Shearn CT, Reigan P, Petersen DR (2012) Inhibition of hydrogen peroxide signaling by 4-hydroxynonenal due to differential regulation of Akt1 and Akt2 contributes to decreases in cell survival and proliferation in hepatocellular carcinoma cells. Free Radic Biol Med 53: 1–11. 22580126 10.1016/j.freeradbiomed.2012.04.021 PMC3377776

[18] Chen J, Wang L, Chen Y, Sternberg P, Cai J (2009) Phosphatidylinositol 3 kinase pathway and 4-hydroxy-2-nonenal-induced oxidative injury in the RPE. Invest Ophthalmol Vis Sci 50: 936–942. 18806289 10.1167/iovs.08-2439 PMC2716057

[19] Yun MR, Im DS, Lee SJ, Woo JW, Hong KW, et al (2008) 4-hydroxynonenal contributes to macrophage foam cell formation through increased expression of class A scavenger receptor at the level of translation. Free Radic Biol Med 45: 177–183. 18456003 10.1016/j.freeradbiomed.2008.04.014

[20] Pope AJ, Druhan L, Guzman JE, Forbes SP, Murugesan V, et al (2007) Role of DDAH-1 in lipid peroxidation product-mediated inhibition of endothelial NO generation. Am J Physiol Cell Physiol 293: C1679–C1686. 17881609 10.1152/ajpcell.00224.2007

[21] Forbes SP, Druhan LJ, Guzman JE, Parinandi N, Zhang L, et al (2008) Mechanism of 4-HNE mediated inhibition of hDDAH-1: implications in no regulation. Biochemistry 47: 1819–1826. 18171027 10.1021/bi701659n

[22] Lin KY, Ito A, Asagami T, Tsao PS, Adimoolam S, et al (2002) Impaired nitric oxide synthase pathway in diabetes mellitus: role of asymmetric dimethylarginine and dimethylarginine dimethylaminohydrolase. Circulation 106: 987–992. 12186805 10.1161/01.cir.0000027109.14149.67

[23] Ambros V (2004) The functions of animal microRNAs. Nature 431: 350–355. 15372042 10.1038/nature02871

[24] Weber M, Baker MB, Moore JP, Searles CD (2010) MiR-21 is induced in endothelial cells by shear stress and modulates apoptosis and eNOS activity. Biochem Biophys Res Commun 393: 643–648. 20153722 10.1016/j.bbrc.2010.02.045 PMC3717387

[25] Wang S, Hu CP, Jiang DJ, Peng J, Zhou Z, et al (2009) All-trans retinoic acid inhibits cobalt chloride-induced apoptosis in PC12 cells: role of the dimethylarginine dimethylaminohydrolase/asymmetric dimethylarginine pathway. J Neurosci Res 87: 1938–1946. 19156866 10.1002/jnr.21999

[26] Zhang X, Ng WL, Wang P, Tian L, Werner E, et al (2012) MicroRNA-21 modulates the levels of reactive oxygen species by targeting SOD3 and TNFalpha. Cancer Res 72: 4707–4713. 22836756 10.1158/0008-5472.CAN-12-0639 PMC3445705

[27] Kaida Y, Ueda S, Yamagishi S, Nakayama Y, Ando R, et al (2012) Proteinuria elevates asymmetric dimethylarginine levels via protein arginine methyltransferase-1 overexpression in a rat model of nephrotic syndrome. Life Sci 91: 301–305. 22749861 10.1016/j.lfs.2012.06.015

[28] Rodionov RN, Murry DJ, Vaulman SF, Stevens JW, Lentz SR (2010) Human alanine-glyoxylate aminotransferase 2 lowers asymmetric dimethylarginine and protects from inhibition of nitric oxide production. J Biol Chem 285: 5385–5391. 20018850 10.1074/jbc.M109.091280 PMC2820767

[29] Strobel J, Mieth M, Endress B, Auge D, Konig J, et al (2012) Interaction of the cardiovascular risk marker asymmetric dimethylarginine (ADMA) with the human cationic amino acid transporter 1 (CAT1). J Mol Cell Cardiol 53: 392–400. 22705145 10.1016/j.yjmcc.2012.06.002

[30] Ishigaki Y, Oka Y, Katagiri H (2009) Circulating oxidized LDL: a biomarker and a pathogenic factor. Curr Opin Lipidol 20: 363–369. 19625960 10.1097/MOL.0b013e32832fa58d

[31] Lee WC, Wong HY, Chai YY, Shi CW, Amino N, et al (2012) Lipid peroxidation dysregulation in ischemic stroke: plasma 4-HNE as a potential biomarker? Biochem Biophys Res Commun 425: 842–847. 22898049 10.1016/j.bbrc.2012.08.002

[32] Yun MR, Park HM, Seo KW, Lee SJ, Im DS, et al (2010) 5-Lipoxygenase plays an essential role in 4-HNE-enhanced ROS production in murine macrophages via activation of NADPH oxidase. Free Radic Res 44: 742–750. 20370567 10.3109/10715761003758122

[33] Lee SJ, Kim CE, Seo KW, Kim CD (2010) HNE-induced 5-LO expression is regulated by NF-{kappa}B/ERK and Sp1/p38 MAPK pathways via EGF receptor in murine macrophages. Cardiovasc Res 88: 352–359. 20554538 10.1093/cvr/cvq194

[34] Ling M, Li Y, Xu Y, Pang Y, Shen L, et al (2012) Regulation of miRNA-21 by reactive oxygen species-activated ERK/NF-kappaB in arsenite-induced cell transformation. Free Radic Biol Med 52: 1508–1518. 22387281 10.1016/j.freeradbiomed.2012.02.020

[35] Strobel J, Mieth M, Endress B, Auge D, Konig J, et al (2012) Interaction of the cardiovascular risk marker asymmetric dimethylarginine (ADMA) with the human cationic amino acid transporter 1 (CAT1). J Mol Cell Cardiol 53: 392–400. 22705145 10.1016/j.yjmcc.2012.06.002

[36] Closs EI, Ostad MA, Simon A, Warnholtz A, Jabs A, et al (2012) Impairment of the extrusion transporter for asymmetric dimethyl-L-arginine: a novel mechanism underlying vasospastic angina. Biochem Biophys Res Commun 423: 218–223. 22609206 10.1016/j.bbrc.2012.05.044

